# Supplementary material for: Developmental and housekeeping transcriptional programs display distinct modes of enhancer-enhancer cooperativity in Drosophila
Source: Nat Commun. 2024 Oct 3;15:8584. doi: 10.1038/s41467-024-52921-2 (PMC11450171; doi:10.1038/s41467-024-52921-2)
Supplement: Supplementary file 1 — Supplementary Information [file 41467_2024_52921_MOESM1_ESM.pdf]

Supplementary Information

Developmental and housekeeping transcriptional programs display distinct modes of enhancer-enhancer cooperativity in *Drosophila*

Vincent Loubiere[1], Bernardo P. de Almeida[1,2], Michaela Pagani[1], Alexander Stark[1,3,*]

[1] Research Institute of Molecular Pathology (IMP), Vienna BioCenter (VBC), Vienna, Austria.

[2] Vienna BioCenter PhD Program, Doctoral School of the University of Vienna and Medical University of Vienna, Vienna, Austria.

[3] Medical University of Vienna, Vienna BioCenter (VBC), Vienna, Austria.

* Corresponding authors: stark@starklab.org

Supplementary figure 1

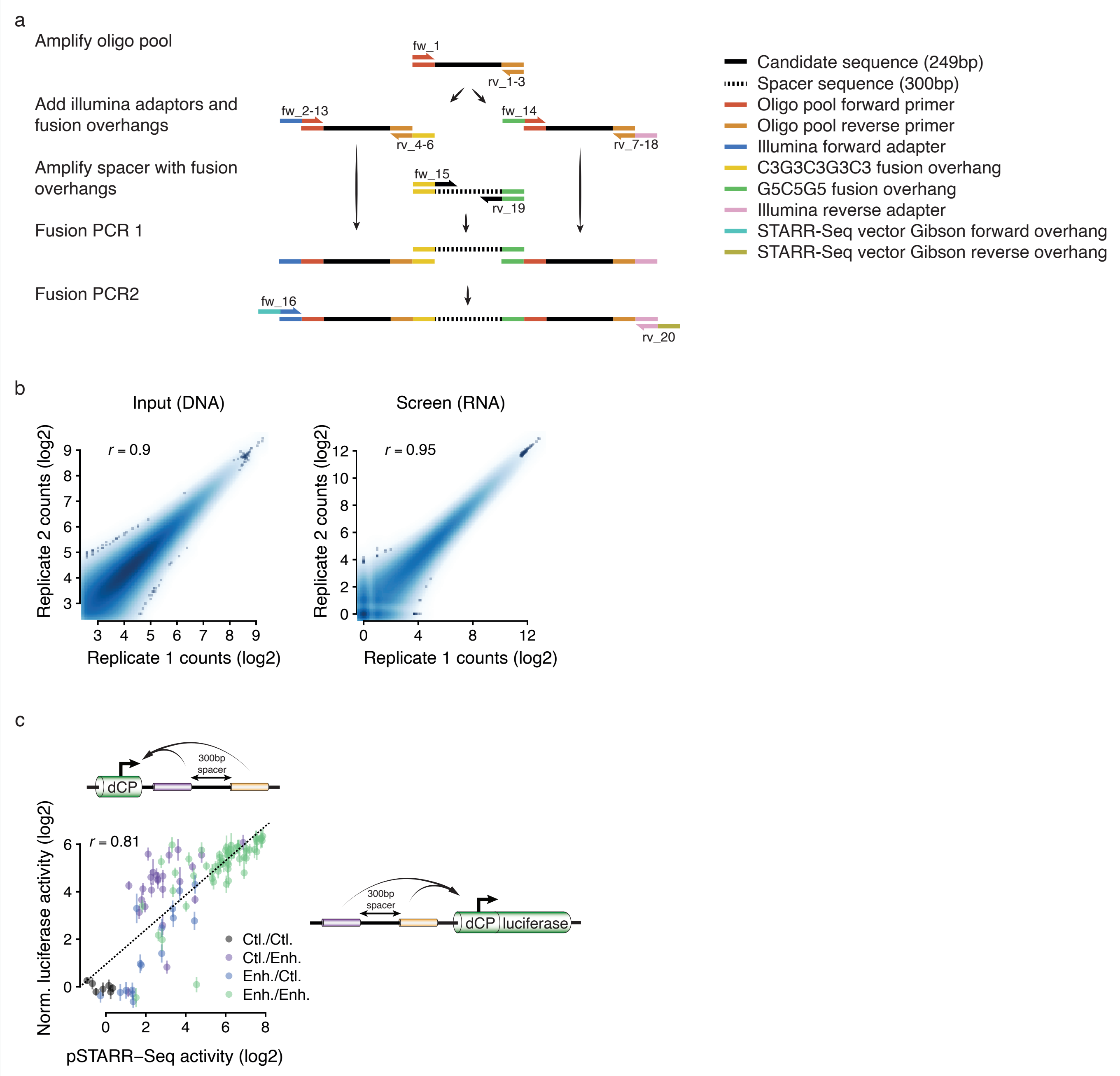

Supplementary Figure 1: Experimental design and luciferase validations

a- Schematic view of the fusion PCR protocol used to generate complex pools of enhancer pairs. **b-** Correlation between RNA (screen) and DNA (input) counts between two STARR-seq replicates. Pearson's correlation coefficient (*r*) is shown (top left). **c-** Correlation between STARR-seq (x axis) and luciferase (y axis) measurements for a set of control-control random sequences (Ctl./Ctl., in grey), one control sequence paired with a candidate sequence either in the 5' (Enh./Ctl., in blue) or the 3' (Ctl./Enh., in purple) location, or two enhancer sequences (Enh./Enh., in green). Schematic views of the reporter constructs used for luciferase (top) or STARR-seq assay (right) are shown, as well as the Pearson's correlation coefficient (*r*, on the top left of the scatterplot). Source data are provided as a Source Data file.

Supplementary figure 2

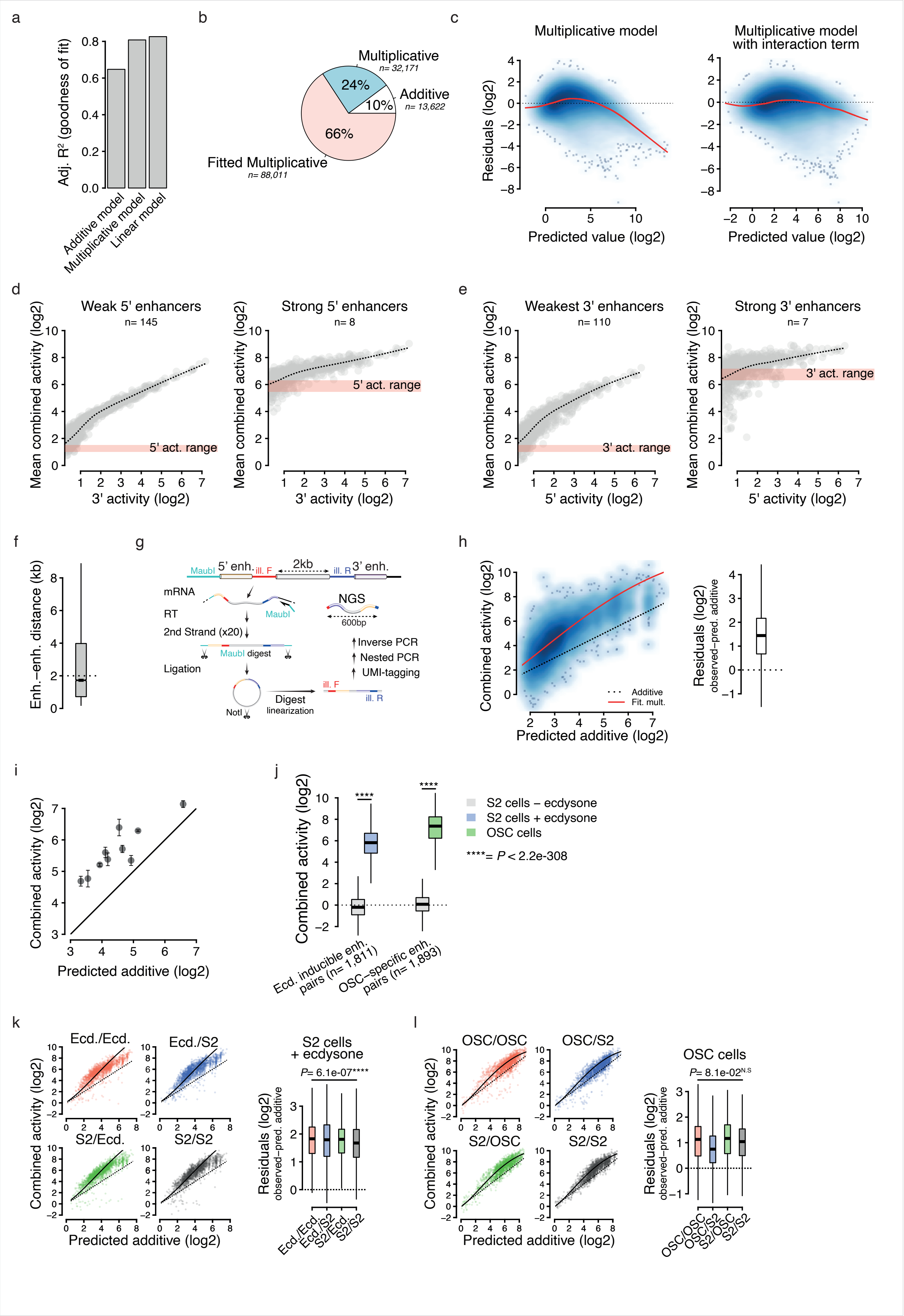

Supplementary Figure 2: Strong developmental enhancer pairs saturate the CP

a- R-squared (R^2) values for the three models (bottom) in the developmental setup (developmental enhancer pairs downstream of a developmental CP). **b-** Fraction of developmental enhancer pairs (in which both candidate sequences are active) for which the additive (in white), the multiplicative (in blue) or the fitted multiplicative model with interaction term (in pink) were the most accurate. **c-** Residuals (y axis) of the multiplicative model (left) and the fitted multiplicative model with interaction term (right) as a function of the predicted values (x axis). The dotted line represents the identity line (where predicted = observed activity); loess regression is shown in red. **d-** Individual activity of 818 enhancers in the 3' position (x-axis) versus their average combined activities (y axis) when paired with a weak (individual activity between 1 and 1.5; n= 145; left scatterplot) or a strong (individual activity > strongest individual activity – 1; n= 8; right scatterplot) 5' enhancer. Weak 5' enhancers can be strongly boosted by the 3' enhancer, pairs containing strong 5' enhancers plateau around eight. **e-** Same as **d** but considering 825 5' enhancers paired with weak (*n*= 110) or strong (n= 7) 3' enhancers. **f-** Enhancer-enhancer distance inferred from STARR-seq data in S2 cells[17]. *n*= 19,735. **g-** Illustration of the 2kb spacer STARR-seq. A MaubI restriction site is appended to the 3' end of enhancer pairs during RT. After second strand synthesis and MaubI digest, fragments are ligated and re-linearized prior to UMI-tagging, inverse PCR and illumina sequencing. **h-** Predicted additive (x axis) *versus* observed (y axis) combined activities of enhancer pairs in which both candidate sequences are active. Identity line (dotted line) and fitted multiplicative model (solid line) are shown. Residuals are quantified on the right (*n*= 8,284). **i-** Predicted additive (x axis) *versus* observed (y axis) combined activities inferred using luciferase assays for 10 different homotypic enhancer pairs. The identity line is shown. Source data are provided as a Source Data file. **j-** Activity of ecdysone-inducible (left) and OSC-specific (right) enhancers in untreated (grey) and ecdysone treated (blue) S2 cells and OSC cells (green). *P*-values: two-sided Wilcoxon tests. **k-** Separated plots for the four categories shown in Fig. 2f (*n*= 1,811; 1,773; 1,814; 1,721). Identity line (dotted line) and fitted multiplicative model (solid line) are shown. Residuals are quantified on the right. *P*-values: two-sided Wilcoxon tests. **l-** Same as k but related to Fig. 2g. *n*= 1,893; 2,000; 1,872; 1,936.

Supplementary figure 3

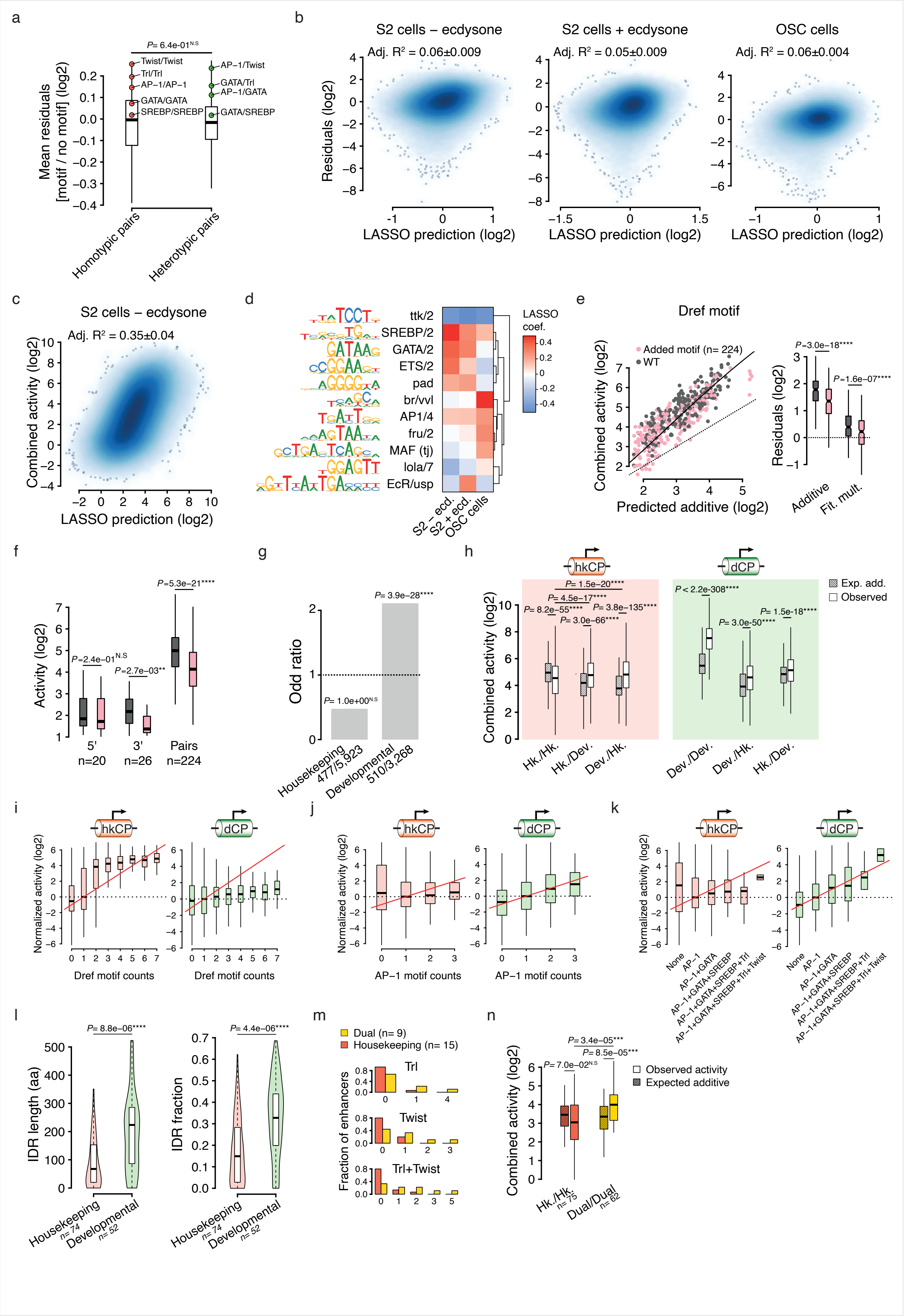

Supplementary Figure 3: Super-additivity does not rely on a rigid motif syntax
a- Mean residual differences from the multiplicative model with interaction for homotypic (left, *n*= 120) *versus* heterotypic (right, *n*= 14,280) TF motif combinations (in which 5' and 3' enhancers contain instance(s) of the same motif or of two different motifs, respectively). Twist/Twist homotypic pairs show higher residuals compared to AP-1/AP-1 pairs. *P*-value: two-sided Wilcoxon test is shown. **b-** Residuals from the multiplicative model with interaction (y axis) as a function of LASSO predictions (x axis) in untreated (left) and ecdysone-treated S2 cells (center) and OSC cells (right). Mean adjusted R-squared (R^2) ± standard deviation (sd) across 9 cross-validation folds are shown. **c-** Combined activities (y axis) as a function of LASSO predictions (x axis). Mean adjusted R-squared (R^2) ± standard deviation (sd) across 9 cross-validation folds is shown. **d-** Top LASSO coefficients in untreated (S2-ecd.) and ecdysone-treated S2 cells (S2+ecd.) and OSC cells (x axis). **e-** Predicted additive (x axis) versus observed (y axis) combined activities of wild-type developmental enhancer pairs (WT, in grey) or after adding Dref motifs (in pink). The additive (dotted line) and the multiplicative model with interaction (solid line) are shown. For both models, WT versus mutant pairs residuals are quantified on the right (see x axis). *P*-values: two-sided Wilcoxon tests. **f-** Impact of adding Dref motifs (in pink) on the individual and combined activities of developmental enhancers. *P*-values: paired, two-sided Wilcoxon tests. **g-** Odd ratios of developmental and housekeeping genes among the top 10% most transcribed genes, defined using PRO-Seq in S2 cells[22]. *P*-values: one-tailed Fisher's exact tests. **h-** Expected additive and observed activities of housekeeping/developmental enhancer pairs (x axis) using either a housekeeping (hkCP, in red) or a developmental (dCP, in green) Core Promoter. *P*-values: two-sided Wilcoxon tests. *n*= 3,590; 2,929; 2,937; 2,328; 2,855; 2,847. **i-** Enhancer activity in S2 cells as a function of Dref motif counts, using a housekeeping (hkCP, in red) or a developmental (dCP, in green) Core Promoter. *n*= 4,146; 365; 789; 162; 339; 56; 85; 41. **j-** Same as h, but for AP-1. *n*= 4,370; 1,312; 249; 52. **k-** Enhancer activity in S2 cells, depending on the presence of at least one motif of each of the factors listed on the x axis, using a housekeeping (hkCP, in red) or a developmental (dCP, in green) Core Promoter. *n*= 1,770; 559; 191; 57; 10; 2. Sequences that contained none of these motifs are marked as "None". **l-** Absolute length (in aa, left) or fraction (right) of Intrinsically Disordered Regions (IDRs) within TF/COF proteins that preferentially activate Housekeeping (Hk., orange) or Developmental (Dev., green) Core Promoters [25]. *P*-values: two-sided Wilcoxon test. **m-** Distribution of Trl (top) Twist (middle) or summed (Trl+Twist) motif counts (x axis) within dual enhancers (in yellow) and an activity-matched set of housekeeping enhancers (orange). **n-** Expected additive and observed activities of housekeeping (Hk./Hk.) and dual (Dual/Dual) enhancer pairs (x axis) using the housekeeping RpS12 CP. *P*-values: two-sided Wilcoxon test are shown.
