# Supplementary material for: Developmental and housekeeping transcriptional programs display distinct modes of enhancer-enhancer cooperativity in Drosophila
Source: Nat Commun. 2024 Oct 3;15:8584. doi: 10.1038/s41467-024-52921-2 (PMC11450171; doi:10.1038/s41467-024-52921-2)
Supplement: Supplementary file 3 — Description of Additional Supplementary Files [file 41467_2024_52921_MOESM3_ESM.pdf]

Description of Additional Supplementary Files

Supplementary Data 1: Oligo pools summary

For each pool, the number of sequences is reported for each enhancer group.

Supplementary Data 2: WT oligo pool sequences

IDs and sequences of the 1,000 oligonucleotide sequences of the WT oligo pool. Flanking PCR primer binding sites are specified.

Supplementary Data 3: PCR primers

PCR primers used to generate enhancer-enhancer pairs using either the 300bp or the 2kb inert spacer. For the 2kb spacer, a schematic view of the method is shown.

Supplementary Data 4: WT oligo pool STARR-seq activities in S2 cells

Individual (Ind) and combined activities (log2FoldChange) of each combination of 5' (L for "Left") and 3' (R for "Right") candidate sequences, measured using the developmental core promoter (DSCP) STARR-seq setup and a 300bp spacer. The "ctlL" and "ctlR" specify whether 5' and 3' sequences were considered as robust controls, respectively. 5' and 3' individual activities are reported in the "indL" and "indR" columns, respectively, with "padjL" and "padjR" columns containing FDR-corrected p-values. These p-values were computed using one-tailed Fisher's exact tests to compare the activities of enhancer/control or control/enhancer pairs against control/control pairs, respectively (only robust controls were used, see Methods). Of note, only the enhancers with an individual activity>1 and a FDR<0.05 are considered active.

Supplementary Data 5: WT oligo pool 2kb spacer STARR-seq activities in S2 cells

Individual (Ind) and combined activities (log2FoldChange) of each combination of 5' (L for "Left") and 3' (R for "Right") candidate sequences, measured using the developmental core promoter (DSCP) STARR-seq setup and a 2kb spacer. The "ctlL" and "ctlR" specify whether 5' and 3' sequences were considered as robust controls, respectively. 5' and 3' individual activities are reported in the "indL" and "indR" columns, respectively, with "padjL" and "padjR" columns containing FDR-corrected p.values. These p-values were computed using one-tailed Fisher's exact tests to compare the activities of enhancer/control or control/enhancer pairs against control/control pairs, respectively (only robust controls were used, see Methods). Of note, only the enhancers with an individual activity>1 and a FDR<0.05 are considered active.

Supplementary Data 6: WT oligo pool STARR-seq activities in S2 cells treated with ecdysone

Individual (Ind) and combined activities (log2FoldChange) of each combination of 5’ (L for “Left”) and 3’ (R for “Right”) candidate sequences, measured using the developmental core promoter (DSCP) STARR-seq setup and a 300bp spacer. The "ctlL" and "ctlR" specify whether 5' and 3' sequences were considered as robust controls, respectively. 5' and 3' individual activities are reported in the "indL" and "indR" columns, respectively, with "padjL" and "padjR" columns containing FDR-corrected p.values. These p-values were computed using one-tailed Fisher's exact tests to compare the activities of enhancer/control or control/enhancer pairs against control/control pairs, respectively (only robust controls were used, see Methods). Of note, only the enhancers with an individual activity>1 and a FDR<0.05 are considered active.

Supplementary Data 7: WT oligo pool STARR-seq activities in OSC cells

Individual (Ind) and combined activities (log2FoldChange) of each combination of 5’ (L for “Left”) and 3’ (R for “Right”) candidate sequences, measured using the developmental core promoter (DSCP) STARR-seq setup and a 300bp spacer. The "ctlL" and "ctlR" specify whether 5' and 3' sequences were considered as robust controls, respectively. 5' and 3' individual activities are reported in the "indL" and "indR" columns, respectively, with "padjL" and "padjR" columns containing FDR-corrected p.values. These p-values were computed using one-tailed Fisher's exact tests to compare the activities of enhancer/control or control/enhancer pairs against control/control pairs, respectively (only robust controls were used, see Methods). Of note, only the enhancers with an individual activity>1 and a FDR<0.05 are considered active.

Supplementary Data 8: List of PWMs used for the design of mutant sequences and motif analyses

Position weight matrices (PWMs) used to count Twist, Trl and Dref motifs for the design of mutated oligos (see Supplementary Table 9) and for the analysis of the residuals (Fig. 3a,b) and LASSO regression (Supplementary Fig. 3b-d). PWMs are reported in the form of log odds.

Supplementary Data 9: Mutated oligo pool sequences

IDs and sequences of the 998 oligonucleotide sequences of the Mutated oligo pool. Flanking PCR primer binding sites are specified.

Supplementary Data 10: Mutated oligo pool STARR-seq activities

Individual (Ind) and combined activities (log2FoldChange) of each combination of 5’ (L for “Left”) and 3’ (R for “Right”) candidate sequences, measured using the developmental core promoter (DSCP) STARR-seq setup and a 300bp spacer. The "ctlL" and "ctlR" specify whether 5' and 3' sequences were considered as robust controls, respectively. 5' and 3' individual activities are reported in the "indL" and "indR" columns, respectively, with "padjL" and "padjR" columns containing FDR-corrected p.values. These p-values were computed using one-tailed Fisher's exact tests to compare the activities of

enhancer/control or control/enhancer pairs against control/control pairs, respectively (only robust controls were used, see Methods). Of note, only the enhancers with an individual activity>1 and a FDR<0.05 are considered active.

Supplementary Data 11: Focused oligo pool sequences

IDs and sequences of the 174 oligonucleotide sequences of the Focused oligo pool. Flanking PCR primer binding sites are specified.

Supplementary Data 12: Focused oligo pool hkCP STARR-seq activities

Individual (Ind) and combined activities (log2FoldChange) of each combination of 5’ (L for “Left”) and 3’ (R for “Right”) candidate sequences, measured using the houskeeping core promoter (RpS12) STARR-seq setup and a 300bp spacer. The "ctlL" and "ctlR" specify whether 5' and 3' sequences were considered as robust controls, respectively. 5' and 3' individual activities are reported in the "indL" and "indR" columns, respectively, with "padjL" and "padjR" columns containing FDR-corrected p.values. These p-values were computed using one-tailed Fisher's exact tests to compare the activities of enhancer/control or control/enhancer pairs against control/control pairs, respectively (only robust controls were used, see Methods). Of note, only the enhancers with an individual activity>1 and a FDR<0.05 are considered active.

Supplementary Data 13: Focused oligo pool dCP STARR-seq activities

Individual (Ind) and combined activities (log2FoldChange) of each combination of 5’ (L for “Left”) and 3’ (R for “Right”) candidate sequences, measured using the developmental core promoter (DSCP) STARR-seq setup and a 300bp spacer. The "ctlL" and "ctlR" specify whether 5' and 3' sequences were considered as robust controls, respectively. 5' and 3' individual activities are reported in the "indL" and "indR" columns, respectively, with "padjL" and "padjR" columns containing FDR-corrected p.values. These p-values were computed using one-tailed Fisher's exact tests to compare the activities of enhancer/control or control/enhancer pairs against control/control pairs, respectively (only robust controls were used, see Methods). Of note, only the enhancers with an individual activity>1 and a FDR<0.05 are considered active.

Supplementary Data 14: Pearson Correlation Coefficients between STARR-seq biological replicates

For each input and screen, Pearson Correlation Coefficients were computed using log2 normalized counts.
