# Supplementary material for: Developmental and housekeeping transcriptional programs display distinct modes of enhancer-enhancer cooperativity in Drosophila
Source: Nat Commun. 2024 Oct 3;15:8584. doi: 10.1038/s41467-024-52921-2 (PMC11450171; doi:10.1038/s41467-024-52921-2)
Supplement: Supplementary file 5 — Reporting Summary [file 41467_2024_52921_MOESM5_ESM.pdf]

nature portfolio

Corresponding author(s): Alexander Stark

Last updated by author(s): Sep 9, 2024

Reporting Summary

Nature Portfolio wishes to improve the reproducibility of the work that we publish. This form provides structure for consistency and transparency in reporting. For further information on Nature Portfolio policies, see our Editorial Policies and the Editorial Policy Checklist.

Statistics

For all statistical analyses, confirm that the following items are present in the figure legend, table legend, main text, or Methods section.

n/a	Confirmed	
☐	☒	The exact sample size (*n*) for each experimental group/condition, given as a discrete number and unit of measurement
☐	☒	A statement on whether measurements were taken from distinct samples or whether the same sample was measured repeatedly
☐	☒	The statistical test(s) used AND whether they are one- or two-sided<br>*Only common tests should be described solely by name; describe more complex techniques in the Methods section.*
☒	☐	A description of all covariates tested
☐	☒	A description of any assumptions or corrections, such as tests of normality and adjustment for multiple comparisons
☐	☒	A full description of the statistical parameters including central tendency (e.g. means) or other basic estimates (e.g. regression coefficient) AND variation (e.g. standard deviation) or associated estimates of uncertainty (e.g. confidence intervals)
☐	☒	For null hypothesis testing, the test statistic (e.g. *F*, *t*, *r*) with confidence intervals, effect sizes, degrees of freedom and *P* value noted<br>*Give P values as exact values whenever suitable.*
☒	☐	For Bayesian analysis, information on the choice of priors and Markov chain Monte Carlo settings
☒	☐	For hierarchical and complex designs, identification of the appropriate level for tests and full reporting of outcomes
☐	☒	Estimates of effect sizes (e.g. Cohen's *d*, Pearson's *r*), indicating how they were calculated

Our web collection on statistics for biologists contains articles on many of the points above.

Software and code

Policy information about availability of computer code

Data collection	For luciferase assays, luciferase and renilla signals were measured using the Gen5 software (Biotek, v3.4) with the synergy H1 plate reader.
Data analysis	All analyses were performed in R (version 4.2.0). The DESeq2 R package was used for differential analysis (v1.38.3) and the glmnet R package for LASSO regression (v4.1-4). All scripts are available at https://github.com/vloubiere/git_peSTARRSeq (DOI: 10.5281/zenodo.13709626).

For manuscripts utilizing custom algorithms or software that are central to the research but not yet described in published literature, software must be made available to editors and reviewers. We strongly encourage code deposition in a community repository (e.g. GitHub). See the Nature Portfolio guidelines for submitting code & software for further information.

Data

Policy information about availability of data

All manuscripts must include a data availability statement. This statement should provide the following information, where applicable:

- Accession codes, unique identifiers, or web links for publicly available datasets
- A description of any restrictions on data availability
- For clinical datasets or third party data, please ensure that the statement adheres to our policy

All raw sequencing data have been deposited on GEO (GSE245033).

Research involving human participants, their data, or biological material

Policy information about studies with human participants or human data. See also policy information about sex, gender (identity/presentation), and sexual orientation and race, ethnicity and racism.

Reporting on sex and gender	No human data was used for this study.
Reporting on race, ethnicity, or other socially relevant groupings	No human data was used for this study.
Population characteristics	No human data was used for this study.
Recruitment	No human data was used for this study.
Ethics oversight	No human data was used for this study.

Note that full information on the approval of the study protocol must also be provided in the manuscript.

Field-specific reporting

Please select the one below that is the best fit for your research. If you are not sure, read the appropriate sections before making your selection.

[x] Life sciences [ ] Behavioural & social sciences [ ] Ecological, evolutionary & environmental sciences

For a reference copy of the document with all sections, see nature.com/documents/nr-reporting-summary-flat.pdf

Life sciences study design

All studies must disclose on these points even when the disclosure is negative.

Sample size	No statistical method was used to predetermine sample size, which were chosen based on the required number of reads to guarantee sufficient sequencing depth, as is critical for the reliable interpretation of STARR-Seq. STARR-Seq assays were always performed in biological duplicates (using independent electroporations on different days), complying with the guidelines referenced in doi: 10.1002/cpmb.105. As such, downstream statistical tests rely on large samples. For luciferase assays, each sample was measured using at least three biological replicates (distinct transfection reactions on different days), with at least three technical replicates each, to ensure the stabilization of the data's central tendency.
Data exclusions	Randomly selected negative sequences that were eventually showing enhancer activity were discarded (|z-score|<1).
Replication	All MPRA experiments were performed using at least two biological replicates (made using independent electroporations at different days) and Pairwise Correlation Coefficients were systematically computed to assess reproducibility (using log2 normalized counts). PCC were high for most samples (≥0.87) and acceptable for the mutant library input (0.83) and the screen with a 2kb spacer (0.67, see Supplementary Table 14). Luciferase measurements relied on at least 3 biological replicates (distinct transfection reactions on different days), with at least 3 technical replicates each (Mean ± Standard Deviation is reported to provide a transparent representation of the low variability of the data).
Randomization	Not relevant for the study (all possible combinations were measured for each library).
Blinding	Blinding was not relevant since all measurements were normalized using internal controls (complying with the gold standards of MPRA techniques) and no qualitative measurements were used.

Reporting for specific materials, systems and methods

We require information from authors about some types of materials, experimental systems and methods used in many studies. Here, indicate whether each material, system or method listed is relevant to your study. If you are not sure if a list item applies to your research, read the appropriate section before selecting a response.

Materials & experimental systems

n/a	Involved in the study	
x		Antibodies
	x	Eukaryotic cell lines
x		Palaeontology and archaeology
x		Animals and other organisms
x		Clinical data
x		Dual use research of concern
x		Plants

Methods

n/a	Involved in the study	
x		ChIP-seq
x		Flow cytometry
x		MRI-based neuroimaging

Eukaryotic cell lines

Policy information about cell lines and Sex and Gender in Research

Cell line source(s)	Drosophila Schneider 2 cells (S2 cells) were obtained from ThermoFisher Scientific (cat. no. R69007). Drosophila OSC cells were obtained from DGRC (stock 288).
Authentication	As the cell lines were purchased form Life Technologies / Thermo Fisher Scientific or DGRC,, visual inspection was used to confirm the morphology.
Mycoplasma contamination	Cell lines were regularly tested negative for mycoplasma.
Commonly misidentified lines (See ICLAC register)	No commonly misidentified cell lines were used in this study.

Plants

Seed stocks	*Report on the source of all seed stocks or other plant material used. If applicable, state the seed stock centre and catalogue number. If plant specimens were collected from the field, describe the collection location, date and sampling procedures.*
Novel plant genotypes	*Describe the methods by which all novel plant genotypes were produced. This includes those generated by transgenic approaches, gene editing, chemical/radiation-based mutagenesis and hybridization. For transgenic lines, describe the transformation method, the number of independent lines analyzed and the generation upon which experiments were performed. For gene-edited lines, describe the editor used, the endogenous sequence targeted for editing, the targeting guide RNA sequence (if applicable) and how the editor was applied.*
Authentication	*Describe any authentication procedures for each seed stock used or novel genotype generated. Describe any experiments used to assess the effect of a mutation and, where applicable, how potential secondary effects (e.g. second site T-DNA insertions, mosiacism, off-target gene editing) were examined.*
